# Supplementary material for: The potential impacts of exploitation on the ecological roles of fish species targeted by fisheries: A multifunctional perspective
Source: PLoS One. 2024 Oct 29;19(10):e0308602. doi: 10.1371/journal.pone.0308602 (PMC11521253; doi:10.1371/journal.pone.0308602)
Supplement: S1 Table — (DOCX) [file pone.0308602.s001.docx]

**S1 Table.** List of fish species reported in the Azorean landings.

| **Code** | **Taxa** | **Code** | **Taxa** |
| --- | --- | --- | --- |
| wah | *Acanthocybium solandri* | mon | *Lophius piscatorius* |
| pho | *Alepocephalus spp* | rng | *Macrouridae* |
| bth | *Alopias superciliosus* | bum | *Makaira nigricans* |
| ele | *Anguilla anguilla* | cbc | *Molva macrophthalma* |
| ahn | *Anthias anthias* | rib | *Mora moro* |
| bsf | *Aphanopus carbo* | mur | *Mullus surmuletus* |
| ogt | *Apogon imberbis* | mwk | *Muraena augusti* |
| blt | *Auxis rochei* | mmh | *Muraena helena* |
| trg | *Balistes capriscus* | mkf | *Mycteroperca fusca* |
| gar | *Belone belone* | sba | *Pagellus acarne* |
| bxd | *Beryx decadactylus* | sbr | *Pagellus bogaraveo* |
| bys | *Beryx splendens* | rpg | *Pagrus pagrus* |
| ivd | *Bodianus scrofa* | gfb | *Phycis blennoides* |
| bog | *Boops boops* | for | *Phycis phycis* |
| oub | *Bothus podas* | flx | *Pleuronectifomes* |
| poa | *Brama brama* | pxv | *Polymixia nobilis* |
| rub | *Caranx crysos* | wrf | *Polyprion americanus* |
| ehi | *Centracanthus cirrus* | bgr | *Pomadasys incisus* |
| gup | *Centrophorus granulosus* | blu | *Pomatomus saltatrix* |
| guq | *Centrophorus squamosus* | poi | *Pontinus kuhlii* |
| cyw | *Centroscymnus owstonii* | bsh | *Prionace glauca* |
| cyp | *Centroselachus crepidater* | prp | *Promethichthys prometheus* |
| gur | *Chelidonichthys cuculus* | trz | *Pseudocaranx dentex* |
| gum | *Chelidonichthys obscurus* | rjc | *Raja clavata* |
| mlr | *Chelon labrosus* | oil | *Ruvettus pretiosus* |
| hzl | *Chromis limbata* | bon | *Sarda sarda* |
| coe | *Conger conger* | pil | *Sardina pilchardus* |
| cou | *Coris julis* | sae | *Sardinella maderensis* |
| dol | *Coryphaena hippurus* | slm | *Sarpa salpa* |
| sck | *Dalatias licha* | hdv | *Schedophilus ovalis* |
| jdp | *Dasyatis pastinaca* | vma | *Scomber colias* |
| dca | *Deania calcea* | rse | *Scorpaena scrofa* |
| sdu | *Deania profundorum* | amb | *Seriola dumerili* |
| swa | *Diplodus sargus* | wsa | *Serranus atricauda* |
| ctb | *Diplodus vulgaris* | cbr | *Serranus cabrilla* |
| awn | *Enchelycore anatina* | bij | *Similiparma lurida* |
|  |  |  | Continue on next page |
|  |  |  |  |
| **Code** | **Taxa** | **Code** | **Taxa** |
| gpd | *Epinephelus marginatus* | prr | *Sparisoma cretense* |
| ggt | *Gaidropsarus guttatus* | bvv | *Sphyraena viridensis* |
| gag | *Galeorhinus galeus* | spz | *Sphyrna zygaena* |
| agk | *Gymnothorax unicolor* | yfw | *Symphodus caeruleus* |
| brf | *Helicolenus dactylopterus* | sdr | *Synodus saurus* |
| hxt | *Heptranchias perlo* | tmp | *Thalassoma pavo* |
| sbl | *Hexanchus griseus* | alb | *Thunnus alalunga* |
| ory | *Hoplostethus atlanticus* | yft | *Thunnus albacares* |
| sma | *Isurus oxyrinchus* | bet | *Thunnus obesus* |
| whm | *Kajikia albida* | bft | *Thunnus thynnus* |
| skj | *Katsuwonus pelamis* | pop | *Trachinotus ovatus* |
| kyp | *Kyphosus spp* | jaa | *Trachurus picturatus* |
| usb | *Labrus bergylta* | swo | *Xiphias gladius* |
| usi | *Labrus mixtus* | jos | *Zenopsis conchifer* |
| sfs | *Lepidopus caudatus* | jod | *Zeus faber* |
| meg | *Lepidorhombus whiffiagonis* |  |  |
