# Supplementary material for: The potential impacts of exploitation on the ecological roles of fish species targeted by fisheries: A multifunctional perspective
Source: PLoS One. 2024 Oct 29;19(10):e0308602. doi: 10.1371/journal.pone.0308602 (PMC11521253; doi:10.1371/journal.pone.0308602)

**S2 Table.** Comparison of functional diversity metrics between local and coastal fishing for fish landed in the Azores archipelago. nbsp: number of species, sing.sp: species functionally different in the landings, quali.FRic: quality of the reduced-space representation required to compute FRic and FDiv, FRic: functional richness, FEve: functional evenness, FDiv: functional divergence, FDis: functional dispersion. P-values represent significance between local and costal pair for each metric in each analysis from randomization testing, considering combined trait modalities (habitat use, locomotion, feeding and life history).


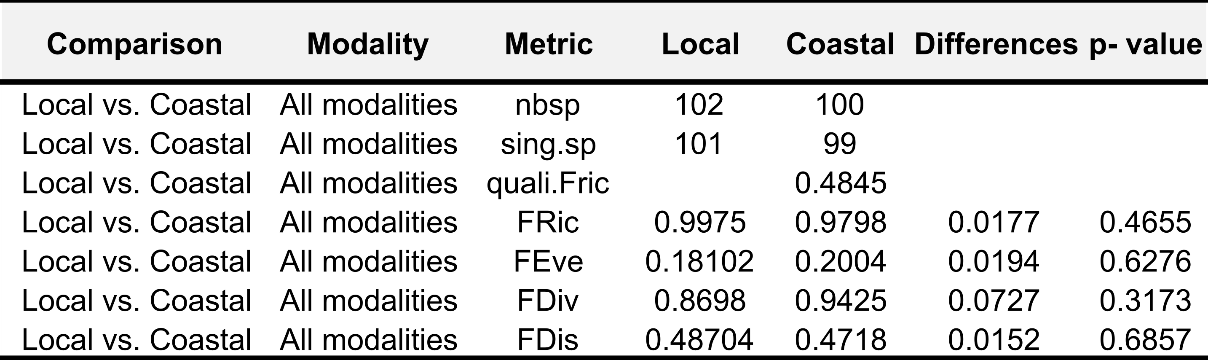

Supplement: S2 Table — nbsp: number of species, sing.sp: species functionally different in the landings, quali.FRic: quality of the reduced-space representation required to compute FRic and FDiv, FRic: functional richness, FEve: functional evenness, FDiv: functional divergence, FDis: functional dispersion. P-values represent significance between local and costal pair for each metric in each analysis from randomization testing, considering combined trait modalities (habitat use, locomotion, feeding and life history). (DOCX) [file pone.0308602.s002.docx]
