# Supplementary material for: The potential impacts of exploitation on the ecological roles of fish species targeted by fisheries: A multifunctional perspective
Source: PLoS One. 2024 Oct 29;19(10):e0308602. doi: 10.1371/journal.pone.0308602 (PMC11521253; doi:10.1371/journal.pone.0308602)

**S3 Table.** Pairwise comparison of functional diversity metrics across functional modalities and decades for species landed by local fishing in the Azores archipelago. FRic: functional richness, FEve: functional evenness, FDiv: functional divergence, FDis: functional dispersion. Significant p-values (p<0.05) are highlighted in red, while marginally significant values are shown in orange. Differences refers to the differences between the observed and the mean values derived from the simulated distribution.


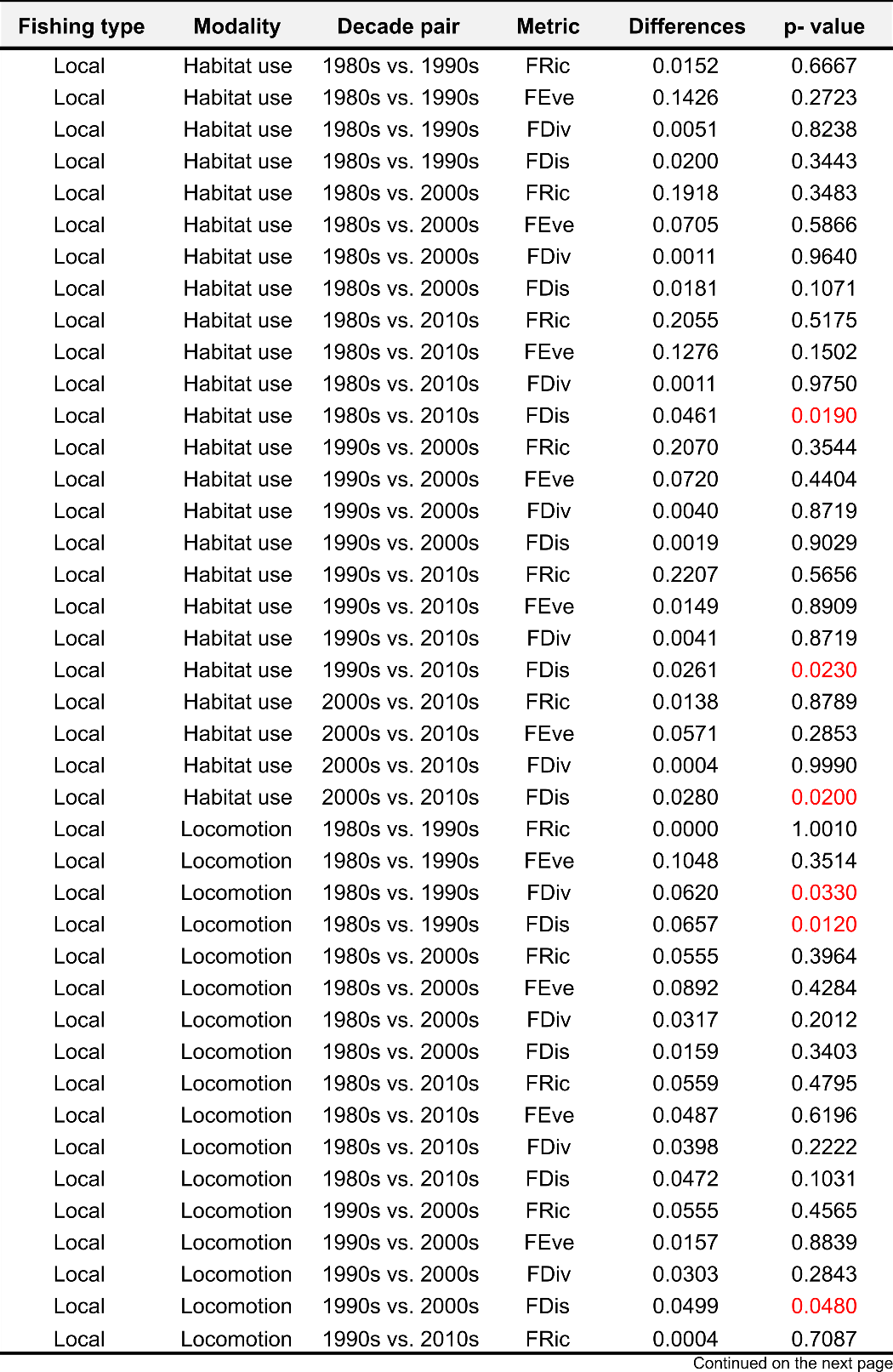


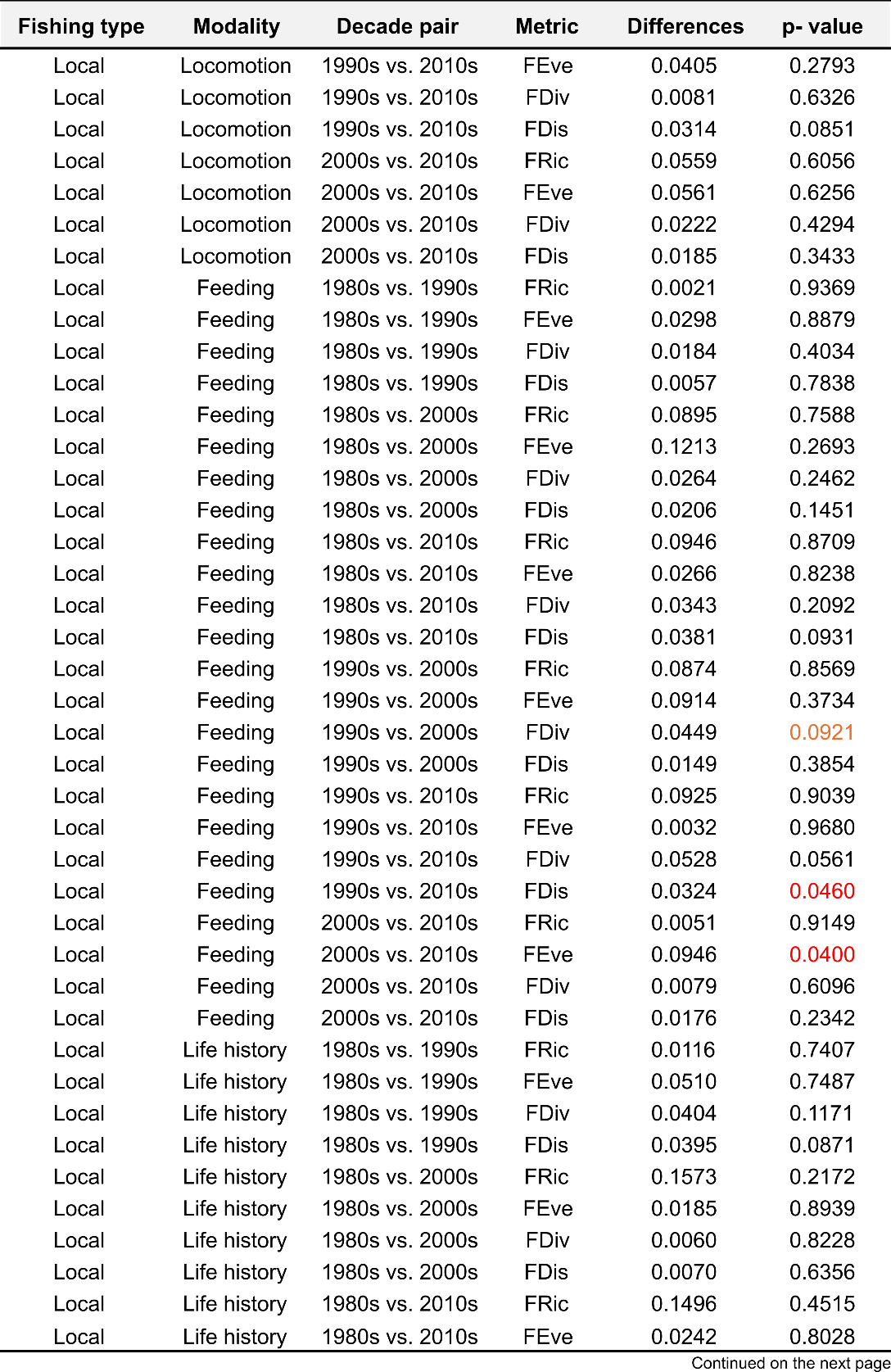


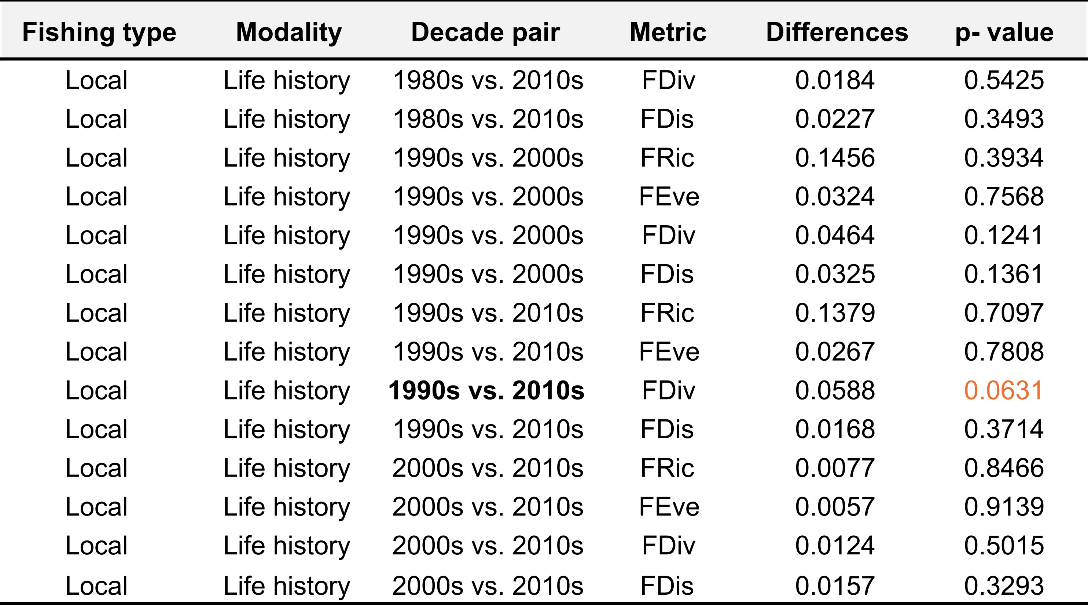

Supplement: S3 Table — FRic: functional richness, FEve: functional evenness, FDiv: functional divergence, FDis: functional dispersion. Significant p-values (p<0.05) are highlighted in red, while marginally significant values are shown in orange. Differences refers to the differences between the observed and the mean values derived from the simulated distribution. (DOCX) [file pone.0308602.s003.docx]
