# Supplementary material for: The potential impacts of exploitation on the ecological roles of fish species targeted by fisheries: A multifunctional perspective
Source: PLoS One. 2024 Oct 29;19(10):e0308602. doi: 10.1371/journal.pone.0308602 (PMC11521253; doi:10.1371/journal.pone.0308602)

**S7 Table.** Pearson correlation (*r^2^*) between the axes of the fuzzy correspondence analysis (FCA) and functional trait categories for feeding modality. Correlations higher than 0.50 and lower than -0.50 are highlighted in green and red, respectively.


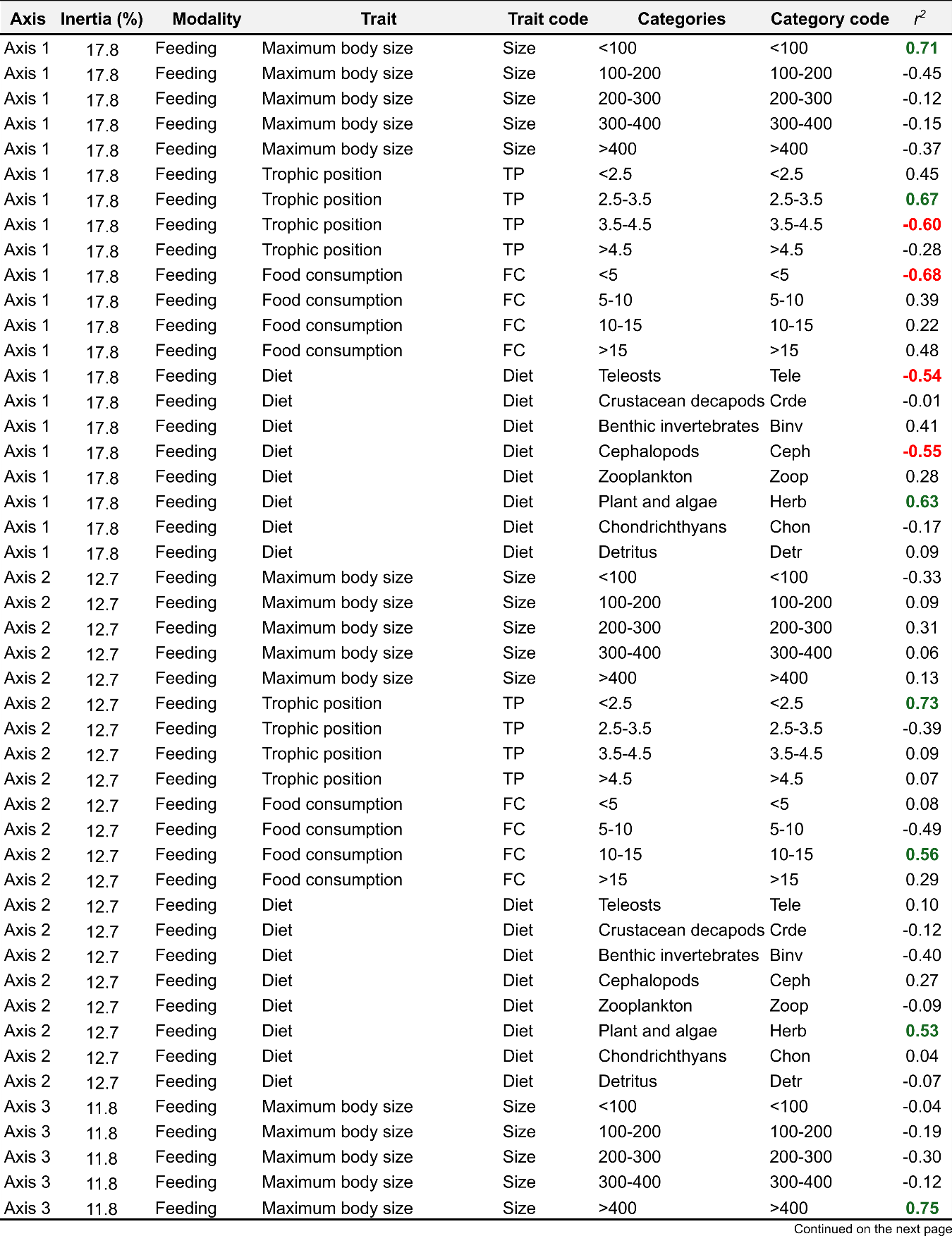


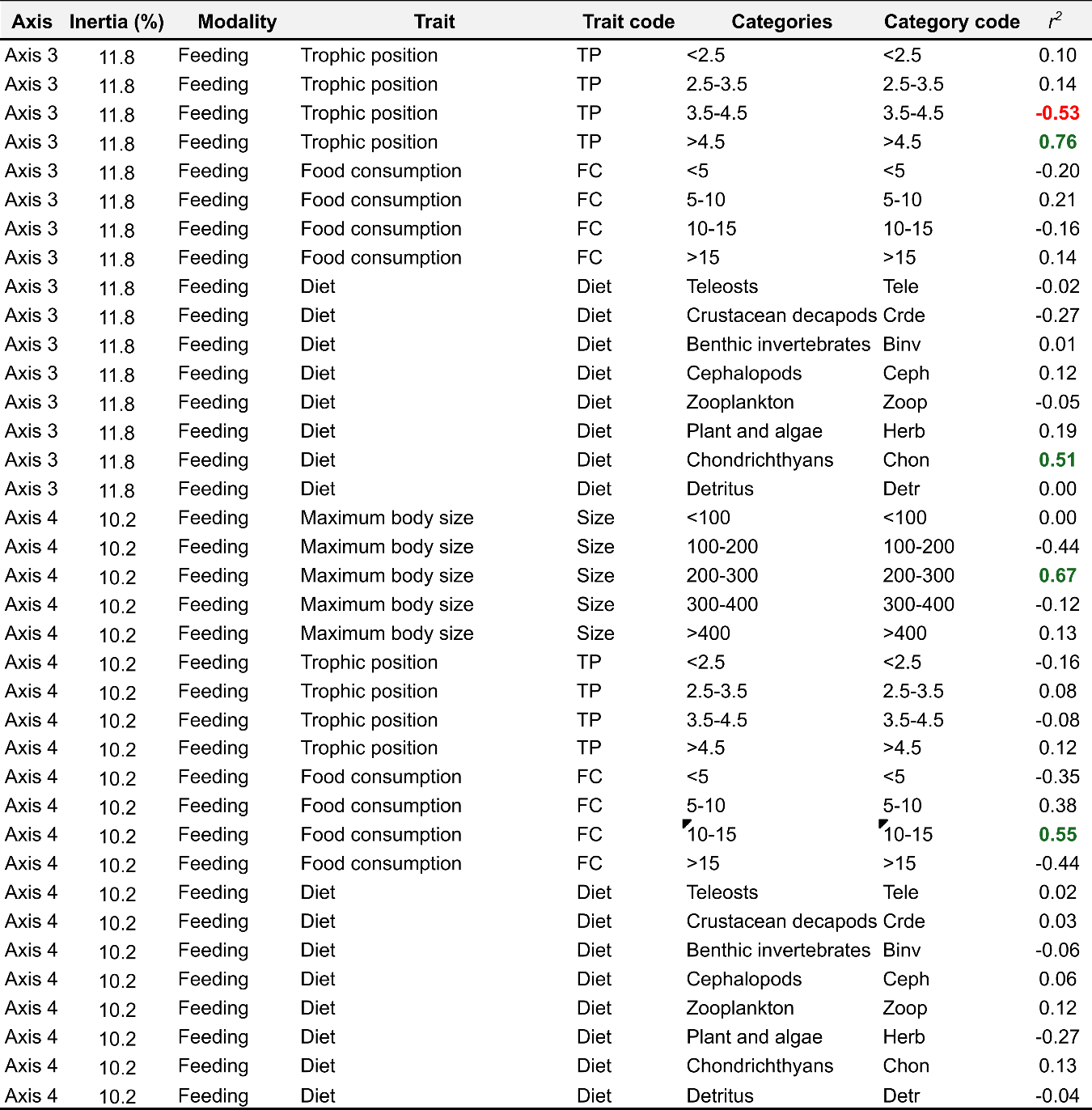

Supplement: S7 Table — Correlations higher than 0.50 and lower than -0.50 are highlighted in green and red, respectively. (DOCX) [file pone.0308602.s007.docx]
