# Supplementary material for: The potential impacts of exploitation on the ecological roles of fish species targeted by fisheries: A multifunctional perspective
Source: PLoS One. 2024 Oct 29;19(10):e0308602. doi: 10.1371/journal.pone.0308602 (PMC11521253; doi:10.1371/journal.pone.0308602)
Supplement: S9 Table — (DOCX) [file pone.0308602.s009.docx]

**S9 Table.** List of species positioned at the vertices of the functional spaces, possessing the most unusual combination of traits.

| **Codes and species names** | **Group** |
| --- | --- |
| **wah** *Acanthocybium solandri* | Actinopterygii |
| **bsf** *Aphanopus carbo* | Actinopterygii |
| **gur** *Chelidonichthys cuculus* | Actinopterygii |
| **gum** *C. obscurus* | Actinopterygii |
| **hzl** *Chromis limbata* | Actinopterygii |
| **dol** *Coryphaena hippurus* | Actinopterygii |
| **awn** *Enchelycore anatina* | Actinopterygii |
| **whm** *Kajikia albida* | Actinopterygii |
| **kyp** *Kyphosus* spp. | Actinopterygii |
| **mon** *Lophius piscatorius* | Actinopterygii |
| **rng** Macrouridae | Actinopterygii |
| **rib** *Mora moro* | Actinopterygii |
| **mwk** *Muraena augusti* | Actinopterygii |
| **blu** *Pomatomus saltatrix* | Actinopterygii |
| **oil** *Ruvettus pretiosus* | Actinopterygii |
| **sae** *Sardinella maderensis* | Actinopterygii |
| **slm** *Sarpa salpa* | Actinopterygii |
| **wsa** *Serranus atricauda* | Actinopterygii |
| **hdv** *Schedophilus ovalis* | Actinopterygii |
| **bij** *Similiparma lurida* | Actinopterygii |
| **yfw** *Symphodus caeruleus* | Actinopterygii |
| **tmp** *Thalassoma pavo* | Actinopterygii |
| **yft** *Thunnus albacares* | Actinopterygii |
| **jod** *Zeus faber* | Actinopterygii |
| **bth** *Alopias superciliosus* | Elasmobranchii |
| **gup** *Centrophorus granulosus* | Elasmobranch |
| **jdp** *Dasyatis pastinaca* | Elasmobranchii |
| **gag** *Galeorhincus galeus* | Elasmobranchii |
| **bsh** *Prionace glauca* | Elasmobranchii |
| **rjc** *Raja clavata* | Elasmobranchii |
| **sbl** *Hexanchus griseus* | Elasmobranchii |
| **sma** *Isurus oxyrinchus* | Elasmobranchii |
| **spz** *Sphyrna zygaena* | Elasmobranchii |
| **cyw** *Centroscymnus owstonii* | Elasmobranchii |
