# Supplementary material for: The potential impacts of exploitation on the ecological roles of fish species targeted by fisheries: A multifunctional perspective
Source: PLoS One. 2024 Oct 29;19(10):e0308602. doi: 10.1371/journal.pone.0308602 (PMC11521253; doi:10.1371/journal.pone.0308602)
Supplement: S1 Fig — (DOCX) [file pone.0308602.s010.docx]

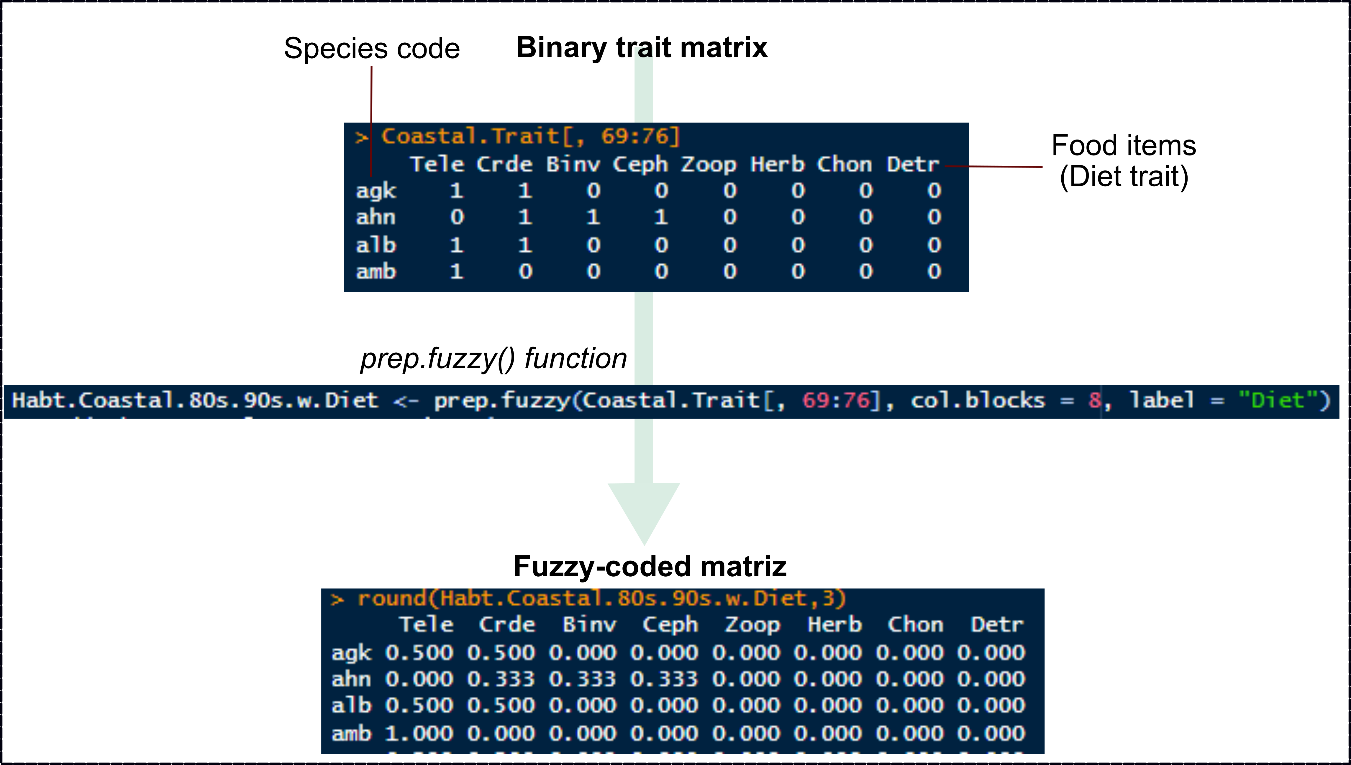


**S1 figure**. Illustration of diet data transformation for four species, demonstrating the data transformation from categorical binary to fuzzy coding.
