# Supplementary material for: The potential impacts of exploitation on the ecological roles of fish species targeted by fisheries: A multifunctional perspective
Source: PLoS One. 2024 Oct 29;19(10):e0308602. doi: 10.1371/journal.pone.0308602 (PMC11521253; doi:10.1371/journal.pone.0308602)
Supplement: S2 Fig — The plots of the fuzzy correspondence analysis (FCA), .i.e., A, C, E and G, illustrate the correlation between the FCA axes (axes 3 and 4), and functional trait modalities (See also S4–S7 Tables and S1 Fig). Different colors represent various trait modalities in FCA plots. In the plots B, D, F, and H, the fifteen most landed species and their total landings in tonnes are highlighted in red. Abbreviation codes available in Table 1. The scientific names of each vertex species highlighted in blue can be seen in S9 Table. (DOCX) [file pone.0308602.s011.docx]

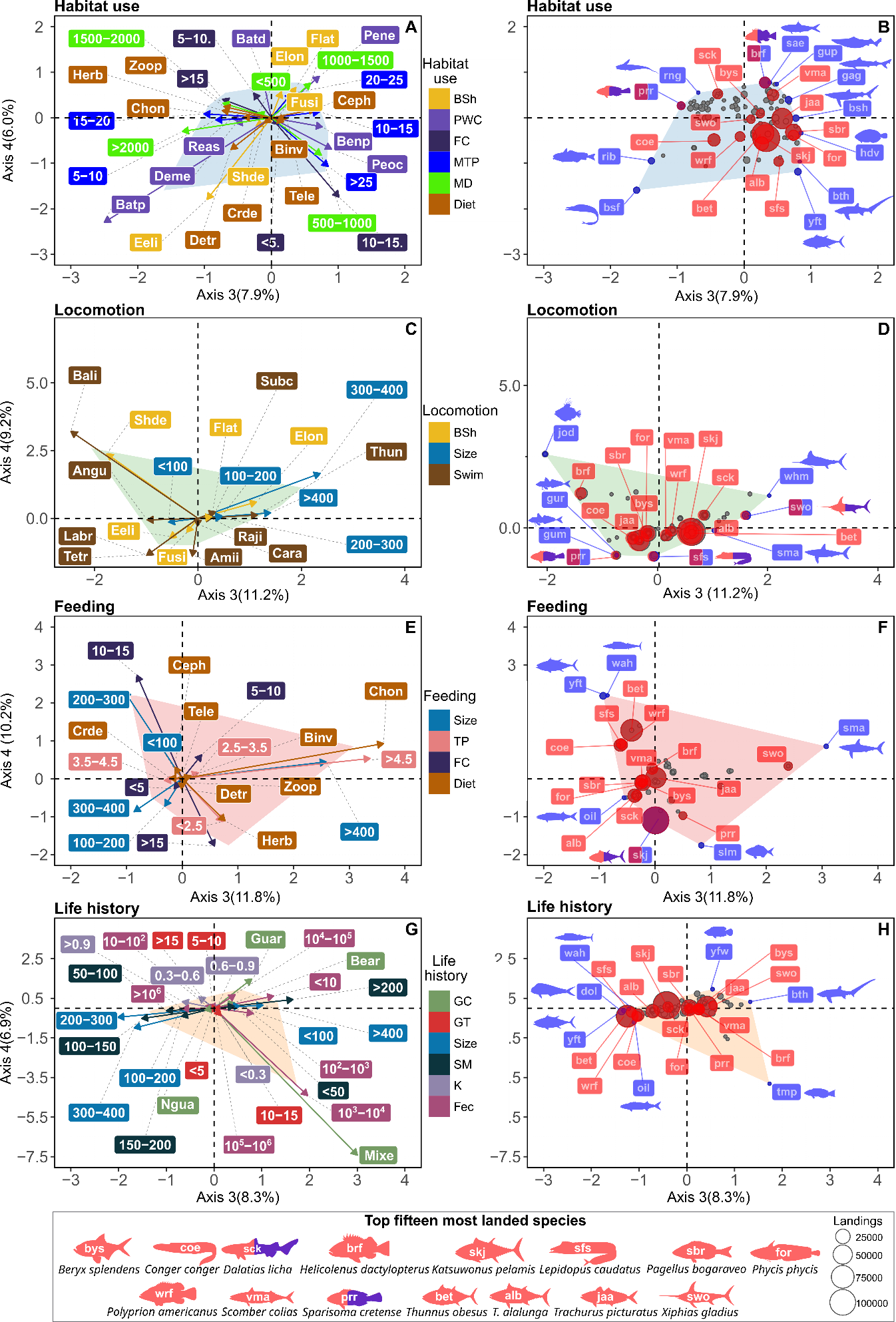


**S2 Figure.** Exploring the relationships between functional traits and fish species within each function (habitat use, locomotion, feeding, and life history) in the functional space (areas in blue, green, light red, and light orange). The plots of the fuzzy correspondence analysis (FCA), .i.e., A, C, E and G, illustrate the correlation between the FCA axes (axes 3 and 4), and functional trait modalities (See also Supplementary Tables 4-7 and Supplementary Figure 1). Different colors represent various trait modalities in FCA plots. In the plots B, D, F, and H, the fifteen most landed species and their total landings in tonnes are highlighted in red. Abbreviation codes available in **Table 1**. The scientific names of each vertex species highlighted in blue can be seen in **Supplementary Table 8**.
