# Supplementary material for: The potential impacts of exploitation on the ecological roles of fish species targeted by fisheries: A multifunctional perspective
Source: PLoS One. 2024 Oct 29;19(10):e0308602. doi: 10.1371/journal.pone.0308602 (PMC11521253; doi:10.1371/journal.pone.0308602)
Supplement: Attachment — Submitted filename: Responses_to_reviewers.docx [file pone.0308602.s012.docx]

Dear Dr. Claudio D'Iglio	July 16, 2024

Academic Editor

PLOS ONE

Dear Editor

First of all, we would like to express our gratitude to you and the reviewers for your thoughtful comments and contributions to the enhancement of our manuscript (manuscript ID: PONE-D-24-22486). We have diligently implemented significant changes and conducted a thorough revision to address all the comments, questions, and suggestions provided by the reviewers. Substantial changes have been made in the Introduction and Discussion sections, including the addition of a subheading in the Discussion that address the limitations of the study and future directions. We also included a supplementary table listing all reported species. Below, we provide detailed responses to each of the reviewers' feedback.

In the files “**response to reviewers**” and “**Revised manuscript with track changes**” you will find:

**In the reviewers’ comments’ file:**

- Reviewers comments in red
- Authors responses in purple
- Changes highlighted in yellow
- Changes based on authors observations are highlight in blue

We look forward to hearing from you at your earliest convenience.

With my best regards,

Dr. Eudriano Costa

Department of Oceanography and Fisheries (IMAR/ OKEANOS)

University of Azores, Horta, Portugal

**RESPONSES TO THE REVIEWER #1**

**General comments: “**The paper is well structured and well written. The tested hypotheses are well defined, as the methods used too. Despite this, a major problem of the paper relies on the lengthy of both introduction and discussion that make the paper difficult to be followed in some parts.”

Response:

Thank you for your positive feedback on the structure and clarity of our paper, as well as on the well-defined hypotheses and methods. We appreciate your constructive criticism regarding the length of the introduction and discussion sections. We reviewed these sections carefully in order to improve readability and coherence. However, we have inserted a new subhead in the Discussion according to reviewer’s #2 suggestions (Please see Lines 634-658).

**Specific comments**

**Comment 1:** “**Concerning all the manuscript**: Please carefully check all the scientific names and use the correct zoological nomenclature, each first time a species is mentioned, e.g. *Pagellus bogaraveo* (Brünnich, 1768). After the first time *P. bogaraveo* can be used. Apply this rule for all the mentioned species. “*Sparissoma cretense*” (line 355)”

Response:

**Line 464**: “…*Conger conger..*.” **was changed** to “…*C. conger..*.”

**Line 464**: “…*Pagellus bogaraveo..*.” **was changed** to “…*P. bogaraveo..*.”

**Line 465**: “…*Helicolenus dactylopterus..*.” **was changed** to “…*H. dactylopterus..*.”

**Line 465**: “…*Phycis phycis..*.” **was changed** to “…*P. phycis..*.”

**Line 485**: “…*Pagellus bogaraveo..*.” **was changed** to “…*P. bogaraveo..*.”

**Line 506**: “…*Pagellus bogaraveo..*.” **was changed** to “…*P. bogaraveo..*.”

**Line 506**: “…*Dalatias licha..*.” **was changed** to “…*D. licha..*.”

**Lines 525-526**: “…*Helicolenus dactylopterus..*.” **was changed** to “…*H. dactylopterus..*.”

**Line 549**: “…*Conger conger..*.” **was changed** to “…*C. conger..*.”

**Line 550**: “…*Raja clavata..*.” **was changed** to “…*R. clavata..*.”

**Line 570**: “…*Conger conger..*.” **was changed** to “…*C. conger..*.”

**Line 570**: “…*Xiphias gladius..*.” **was changed** to “…*X. gladius..*.”

**Lines 620-621**: “…*Helicolenus dactylopterus..*.” **was changed** to “…*H. dactylopterus..*.”

**Line 625**: “…*Galeorhinus galeus..*.” **was changed** to “…*G. galeus..*.”

**Line 626**: “…*Prionace glauca..*.” **was changed** to “…*P. glauca..*.”

**Comment 2:** “**Title**: The title well frames only part of the performed study, and it should be modified to better reflects all the parts of the study. It would be better to highlights, starting from the title, “the potential impacts of exploitation on the ecological roles of fish species targeted by fisheries”. Moreover, it is no clear which traits are focused. Add “functional”?”

Response:

**Lines 1-3. Title**: “Trait diversity in exploited fish of the Azorean islands: Insights from a multifunctional approach” **was changed to** “The potential impacts of exploitation on the ecological roles of fish species targeted by fisheries: a multifunctional perspective”

We believe that the new title better reflects our study, highlighting its relevance and scope, and making it more informative to the target audience. Additionally, the word “multifunctional” indicates that that the study examined various traits associated with different ecological roles, and how these traits may contribute to the overall functioning of the ecosystem.

**Comment 3:** “**Abstract:** Lines 44-47: which findings highlighted the importance of adaptive management strategies? Please briefly state here or re-arrange the abstract conclusion. Keywords: “ecosystem functioning” is a bit off topic. Please consider replacing with another keyword.”

Response:

**Line 45-47**: “The findings highlight the importance of adaptive management strategies to address fishing impacts on species traits and their ecological roles, crucial for long-term fisheries and ecological sustainability.” **was changed** to “The findings highlight the importance of addressing fishing impacts on species traits and their ecological roles, which is crucial for long-term fisheries and ecological sustainability.”

**Keywords**: “ecosystem functioning” **was changed** to “ecological roles”.

**Comment 4:** “**Introduction:** Despite the authors well introduce their study, as already motioned, some parts of the introduction are too lengthy bringing the lecturer to information far from the paper. All the paragraph related to Azores fisheries can be drastically reduced to few lines.”

Response:

The indicted paragraphs were shortened as following below:

**Lines 89-101**: “Fisheries play a vital role in the Azores Archipelago. Despite its rich marine biodiversity, fishing grounds are rare, small, and scattered, with less than 1% of the Exclusive Economic Zone (EEZ) having depths less than 600 m, and an average depth of 3000 m [24,25]. The Azorean fishing industry has expanded since the 1980s from the island shelves to offshore seamount areas and deeper waters. This expansion was facilitated by advancements in air transportation of fresh fish products to mainland Portugal and foreign countries, as well as by the adoption of boats equipped with high-tech equipment such as sonar, which increased the autonomy of fishing vessels [26–28]. Azorean fisheries are classified as small-scale because approximately 60% of the vessels are under nine meters in length [28]. Small scale fishing represents between 80-90% of the Azorean fishing fleet [26,27,29]. This fleet targets a variety of fish species with different economic values, playing different ecological roles at various depths in nearshore and seamounts areas [20,24,28,30,31]. The most important targets are tuna and tuna-like species (in weight), deep-water demersal species (in value), and small pelagic species [28]. These species are landed at Azores Auction services for commercialization by two types of fishing fleets: local and coastal fleets. The former comprises small boats less than 12 meters in length, operating in proximity to coastal areas at depths up to 700 m with limited autonomy, while the latter explores more distant waters deeper than 700 m, focusing on large pelagic and deep-water demersal species [27,32]. Both fleets utilize high selective gears employing hook and line, including a variety of handlines and longlines, which are the most important fishing methods. However, traps and small purse-seine methods may be used by both fleets, but they are more commonly utilized by local fishing operations [27,28].” **was changed** **to** “Fisheries are crucial in the Azores Archipelago, which, despite its rich marine biodiversity, has limited fishing grounds with less than 1% of its EEZ being shallower than 600 m and an average depth of 3000 m [24,25]. Since the 1980s, the Azorean fishing industry has expanded from island shelves to offshore seamount areas and deeper waters, driven by advancements in air transport of fresh fish and high-tech equipment on boats, such as sonar [26–28]. Classified as small-scale, about 60% of Azorean vessels are under nine meters in length, and small-scale fishing comprises 80-90% of the fleet [26,27,28,29]. This fleet targets various species, including tuna, deep-water demersal, and small pelagic species, across different depths [20,24,28,30,31]. Fish are commercialized through local and coastal fleets: the local fleet operates small boats (under 12 meters) near coastal areas up to 700 m deep, while the coastal fleet targets deeper waters over 700 m [27,32]. Both fleets primarily use selective hook and line gears, such as handlines and longlines, with traps and small purse-seine methods also used, especially by local operations [27,28].”

**Lines 102-110**: “The small-scale fisheries in the Azores are recognized for their sustainable practices, supported by an efficient and unique system for fishery data collection established since the 1970s, and local regulation measures [33,34]. These regulations include area and temporal fishing closures (e.g., local regulation ordinance n° 74/2015), species-specific quotas, and minimum landing sizes (e.g., local regulation ordinance n° 21/2019). Furthermore, there are limits for annual landings of many Actinopterygii species, including *Phycis phycis*, *Helicolenus dactylopterus*, *Serranus atricauda*, and *Pagellus bogaraveo*, and , and the forbitten to target two shark species *Galeorhinus galeus* and *Prionace glauca* (e.g., local regulation ordinances n° 92/2019 and n° 27/2023). Despite all efforts to manage and promote the sustainable use of the main fishery resources in Azorean waters, there is still a lack of knowledge regarding the potential impacts of fishing activities on marine biodiversity, ecosystem function, and services.” **was changed** to “The small-scale fisheries in the Azores are recognized for their sustainable practices, supported by an efficient fishery data collection system established since the 1970s and local regulations [33,34]. These regulations include fishing closures (e.g., ordinance n° 74/2015), species-specific quotas, and minimum landing sizes (e.g., ordinance n° 21/2019). Limits are set for annual landings of species like *Phycis phycis*, *Helicolenus dactylopterus*, *Serranus atricauda*, and *Pagellus bogaraveo*, with a ban on targeting sharks *Galeorhinus galeus* and *Prionace glauca* (e.g., ordinances n° 92/2019 and n° 27/2023). Despite efforts for sustainable resource use, knowledge gaps remain regarding the potential impacts of fishing on marine biodiversity and ecosystem functions.”

**Comment 5:** “**Material and methods:** 2.2 “Lading data”  Landing It is necessary to present the list of total fish species as part of this paper. It is unconceivable that a reader has to switch to another paper to see on which species the study is performed. Table 1: Swimming mode  Correct the following: “Thunniform (Tetr)” (Tetr) is already used for Tetraodontiform.”

Response:

**Line 46**: “The list of all fish species landed in Azores, along with the total landings by species, can be found in Costa et al. [20], and Costa et al. [29].” **was changed** to “All fish species included in this study are listed in S1 Table. For more information about all fish species reported in the Azorean landings, as well as the total landings by species, can be found in Costa et al. [20], and Costa et al. [29].”

**Supplementary information: “**S1 Table” **was inserted**.

**Supplementary information: “**S1 Table” **was changed to** “S2 Table”.

**Supplementary information: “**S2 Table” **was changed to** “S3 Table”.

**Supplementary information: “**S3 Table” **was changed to** “S4 Table”.

**Supplementary information: “**S4 Table” **was changed to** “S5 Table”.

**Supplementary information: “**S6 Table” **was changed to** “S7 Table”.

**Supplementary information: “**S7 Table” **was changed to** “S8 Table”.

**Supplementary information: “**S8 Table” **was changed to** “S9 Table”.

**Line 308:** “…from 22.5 % to 30.5 % (**S1 Table**).” **was changed to** “…between the two fishing types (**S2 Table**).”

**Line 336:** “…from 22.5 % to 30.5 % (**S4-S7 Tables**).” **was changed to** “…from 22.5 % to 30.5 % (**S5-S8 Tables**).”

**Line 348:** “…can be found in **S4 Table**.” **was changed to** “…can be found in **S5 Table**.”

**Line 361:** “…FCA locomotion can be found in **S5 Table**.” **was changed to** “…FCA locomotion can be found in **S6 Table**.”

**Line 376:** “…FCA feeding can be found in **S6 Table**.” **was changed to** “…FCA feeding can be found in **S7 Table**.”

**Line 392:** “…FCA life history can be found in **S7 Table**.” **was changed to** “…FCA life history can be found in **S8 Table**.”

**Line 407:** “…**S2 Table and Costa et al. (2024a)**” **was changed to** “…**S3 Table and Costa et al. (2024a)**”.

**Lines 415-416:** “…For details see **S3 Table and Costa et al. (2024a)**.” **was changed to** “…For details see **S4 Table** and Costa et al. (2024a).”

**Lines 422-423:** “…and functional trait modalities (See also S4-7 Tables and S1 Fig).” **was changed to** “…and functional trait modalities (See also **S5-S8 Tables and S1 Fig**).”

**Line 426:** “…highlighted in blue can be seen in **S8 Table**.” **was changed to** “…highlighted in blue can be seen in **S9 Table**.”

**Lines 183-184 - Table 1**: “Thunniform (Tetr)” **was changed** to “Thunniform (Thun)”.

**Comment 6:** “**Discussion:** Discussion section is very detailed, but it needs to be reduced, avoiding redundance of some sentences. This could help in improving readability of the paper and the focus on specific results obtained.”

Response:

Thank you for your feedback. While the reviewer did not specify which paragraphs to shorten or where to reduce redundancy. Nevertheless, we have made adjustments to enhance clarity and conciseness throughout the text. These changes were aimed at maintaining focus and clarity while preserving depth of content. Regarding the topic “4.2 Fuzzy Correspondence Analysis”, we have decided not to shorten this section because we consider its content essential for understanding the potential impacts of fisheries on traits relative to their functions. This analysis provides crucial insights the contribute significantly to the overall comprehension of our study’s findings.

**Lines 442-454**: “Fishing activities have the potential to influence diversity metrics estimation and alter the composition of functional traits within ecosystems over time by modifying both the species composition of catches and the amount of biomass landed [7,15,17,20,71]. Indeed, Costa et al. [20] reported significant variations in FRic, and functional space of fish species landed in the Azores for the last three decades. These authors attributed such changes to variations of species that possessed an unusual combination of traits reported in annual landings. Additionally, Costa et al. [36] found significant changes in species composition landed by both local and coastal fishing in the Azores, highlighting the implications for diversity metric estimation such as functional redundancy (FRed) and functional vulnerability (FVul). However, neither of these studies conducted a comprehensive investigation into trait diversity as performed in the present study using null models. Thus, the present study fills this gap by revealing the absence of significant in FRic between fishing types and over time across functions, suggesting that azorean fisheries has targeted species with the same range of traits through both local and coastal fishing over the past four decades.” **was changed** to “Fishing activities can influence diversity metrics and alter functional traits within ecosystems by changing species composition and biomass landed [7,15,17,20,71]. Costa et al. [20] reported significant variations in FRic and functional space of fish species landed in the Azores over the past three decades, attributed to variations in species with unusual trait combinations in annual landings. Costa et al. [36] also found significant changes in species composition landed by both local and coastal fishing in the Azores, affecting diversity metrics such as functional redundancy (FRed) and functional vulnerability (FVul). However, these studies did not comprehensively investigate trait diversity. The present study fills this gap using null models, revealing no significant differences in FRic between fishing types and over time across functions, suggesting that Azorean fisheries have targeted species with a consistent range of traits through both local and coastal fishing over the past four decades.”.

**Lines 455-473**: “Despite the absence of significant variations in trait diversity across decades, it is important to highlight that species landed by Azorean fishing fleets exhibit a wide range of combination of traits, suggesting that the landed species occupy a diverse array of ecological niches. This holds true even when considering only the fifteen most landed species. In azorean waters, fish are primarily caught using many different types of handlines and longlines. However, small pelagic species such as *Trachurus picturatus*, *Scomber japonicus*, and *Boops boops* are commonly caught using purse-seine nets [27,28,72]. Mbaru et al. [15] has suggested that the selective gear types used target a narrower range of functional diversity, implying that the targeted fish species may not exhibit a high diversity of traits, and potentially impact fewer ecosystem functions. In the Azores, the majority of selective gear types are associated with bottom habitats to capture species such as Raja clavata (Elasmobranchii), *P. bogaraveo*, *C. conger*, *H. dactylopterus*, *Mora moro*, *P. phycis*, *Beryx* spp., among others [72,73]. Menezes et al. [24] reported that the species composition of fish assemblages in the Azores were closely related to depth, identifying three main depth-related ichthyofaunistic discontinuities (<200 m, 200-600 m, and >700 m). Additionally, the type of azorean fishing fleet is also closely related to the specific fishing areas and depths they visit. For instance, coastal fishermen using longlines typically catch fish at deeper waters, usually between 200 and 700 meters, compared to the local fishing fleet [24,32]. Therefore, the disparity between the findings of the current study and that published by Mbaru et al. [15] can be attributed to the species diversity and variability in bathymetric composition of fish species landed in Azorean islands. These factors contributed to the diversity of trait combinations among landing species.” **was changed** to “Despite the absence of significant variations in trait diversity across decades, the species landed by Azorean fishing fleets exhibit a wide range of trait combinations, indicating occupation of diverse ecological niches. This diversity is evident even among the fifteen most landed species. In Azorean waters, fish are primarily caught using various handlines and longlines, while small pelagic species such as *Trachurus picturatus*, *Scomber japonicus*, and *Boops boops* are caught using purse-seine nets [27,28,72]. Mbaru et al. [15] suggested that selective gear types target a narrower range of functional diversity, potentially impacting fewer ecosystem functions. In the Azores, most selective gear types are associated with bottom habitats, capturing species such as *Raja clavata*, *P. bogaraveo*, *C. conger*, *H. dactylopterus*, *Mora moro*, *P. phycis,* and *Beryx* spp. [72,73]. Menezes et al. [24] reported that fish assemblages in the Azores are closely related to depth, with three main depth-related discontinuities (<200 m, 200-600 m, and >700 m). The type of fishing fleet is also related to specific fishing areas and depths, with coastal fishermen using longlines typically catching fish at deeper waters (200-700 meters) compared to the local fishing fleet [24,32]. The disparity between our findings and those of Mbaru et al. [15] can be attributed to the species diversity and variability in bathymetric composition of fish species landed in the Azores, contributing to the diversity of trait combinations among landed species.”

**Line 474-492**: “The amount of landed fish can also influence the types of trait combinations removed from the ecosystem by fisheries over time, and this may be reflected in changes in metrics affected by the catches, such as FEve, FDiv, and FDis [7,18–20]. Although trait diversity did not change over the past 40 years in the Azorean waters, significant differences were identified between some decade pairs in FEve, FDiv and FDis in both coastal and local landings. Exploiting a broader array of species that fulfill diverse ecological roles (resulting in higher trait diversity), while simultaneously increasing in exploitation of specific groups of species, can lead to changes in these metrics [17]. For instance, Costa et al. [20] reported an increase in landings of the species such as Trachurus picturatus, Katsuwonus pelamis and Thunnus obesus, which contributed to a decrease in FEve over the last two decades in Azores. These authors also attributed variability in FDiv to the species K. pelamis, which represented the highest landings and possessed unusual combinations of traits compared to other landed species in recent decades. Additionally, Costa et al. [36] reported differences between fishing types in the ten most landed species in Azores islands in terms of landings. Specifically, for local fishing, the top three most landed species were T. picturatus, P. bogaraveo and K. pelamis, whereas for coastal fishing, they were K. pelamis, T. obesus and T. picturatus. Indeed, it’s common for landings to be dominated by a relatively small number of taxa, which can also affect diversity metrics weighted by catch data [18,19,71,73,74]. Therefore, the significant differences observed in FEve, and FDis between local and coastal fishing across functions, particularly evident in the Figs 3C and 4B, may be attributed to temporal variability in the catches of the most landed species, possessing unusual trait combinations compared to the other landed species.” **was changed** **to** “The amount of landed fish can influence the types of trait combinations removed from the ecosystem by fisheries over time, affecting metrics such as FEve, FDiv, and FDis [7,18–20]. Although trait diversity did not change over the past 40 years in Azorean waters, significant differences were found between some decade pairs in FEve, FDiv, and FDis in both coastal and local landings. Exploiting a broad array of species with diverse ecological roles can change these metrics [17]. Costa et al. [20] reported increased landings of species such as *Trachurus picturatus*, *Katsuwonus pelamis*, and *Thunnus obesus*, contributing to a decrease in FEve over the last two decades in the Azores. Variability in FDiv was also linked to *K. pelamis*, which had unusual trait combinations compared to other landed species in recent decades. Costa et al. [36] noted differences in the ten most landed species between fishing types in the Azores, with *T. picturatus*, *P. bogaraveo*, and *K. pelamis* being most landed by local fishing, while *K. pelamis*, *T. obesus*, and *T. picturatus* dominated coastal fishing. Landings often dominated by a few taxa can impact diversity metrics weighted by catch data [18,19,71,73,74]. Thus, the significant differences observed in FEve and FDis between local and coastal fishing, particularly in Figs 3C and 4B, may result from temporal variability in the catches of the most landed species with unusual trait combinations compared to the other landed species.”

**Line 608-629**: “The seasonal availability and increased catch of specific fish species can lead to the significant removal of traits and functions from ecosystems, impacting ecosystem structure, dynamics, and resilience [1,8,71,78]. By considering the sensitivity of metrics weighted by catch, such as FEve, FDiv, and FDis, fisheries managers can gain a better understanding of and mitigate the ecological impacts of seasonal fishing pressure [7,17–20]. This includes targeting specific species or groups of species by identifying the traits related to the highest landings. Another important point to consider is incorporating the life history traits of targeted species into sustainable management strategies to ensure the long-term viability of fisheries resources. For species with high fecundity and low generation time, management measures such as size limits, catch quotas, and seasonal closures play a crucial role in preventing overexploitation and maintaining sustainable exploitation levels. Species with contrasting life history strategies, such as elasmobranchs, may require more precautionary management approaches, including stricter catch limits, protected areas, and efforts to reduce bycatch. In Azores, for instance, the local regulation ordinance n° 21/2019 play an important role stablishing the minimum size of catch for species such as *H. dactylopterus*, *C. conger*, *P. bogaraveo*, *Raja* spp. (Elasmobranchii), *Beryx* spp., *S. cretense*, and others. Another significant local regulation is ordinance n° 92/2019, which sets limits on annual landings for many species targeted by both fishing local and coastal fleets, including those highlighted in the present study with unusual combinations of traits (vertex species) such as *R. clavata*, and *S. cretense*. This ordinance also prohibits the capture of Elasmobranchii, particularly *Isurus* spp., *G. galeus* and *P. glauca* in Azorean waters. Therefore, it is essential to consider the intraspecific trait variability of exploited fish and their association functions and process over time for adaptive fisheries management, conservation efforts, and maintaining ecosystem functioning and services.” **was changed** **to** “Seasonal fluctuations and increased catches of specific fish species can significantly deplete traits and functions from ecosystems, impacting their structure, dynamics, and resilience [1,8,71,78]. Understanding and mitigating these ecological impacts involves considering metrics weighted by catch, such as FEve, FDiv, and FDis, to better manage seasonal fishing pressures [7,17–20]. This includes targeting species with traits linked to high landings. Incorporating life history traits into sustainable management strategies is crucial for ensuring long-term fisheries viability. For species with high fecundity and short generation times, measures such as size limits, catch quotas, and seasonal closures are essential to prevent overexploitation. Species with different life history strategies, such as elasmobranchs, may require more cautious management, including stricter catch limits, protected areas, and measures to reduce bycatch. In the Azores, regulations like ordinance n° 21/2019 establish minimum catch sizes for species such as *H. dactylopterus*, *C. conger*, *P. bogaraveo*, and various Elasmobranchii species. Ordinance n° 92/2019 sets annual landing limits for many species targeted by local and coastal fleets, including those identified in our study with unique trait combinations (vertex species) such as *R. clavata* and *S. cretense*. This ordinance also prohibits the capture of Elasmobranchii species such as *Isurus* spp., *G. galeus*, and *P. glauca* in Azorean waters. Therefore, considering the intraspecific trait variability of exploited fish and their associated functions over time is essential for adaptive fisheries management, conservation efforts, and maintaining ecosystem functioning and services.”

**Comment 7:** “**Conclusion:** I suggest the authors be more cautious in drawing the conclusions of their study. As the authors state in different points of their paper there is, in the results “the absence of significant variations in trait diversity across decades…”; then, drawing strong conclusions without a historical data base is not possible. Please avoid in the conclusion the use of terms as “valuable”, it is a bit self-aggrandizing.”

Response:

We appreciate the reviewer's feedback and their attention to the caution needed in drawing conclusions from our study. We have taken into account the absence of significant variations in trait diversity across decades, as highlighted in our results. Our conclusions are based on a comprehensive historical dataset spanning 40 years, which provides a robust foundation for our findings. Nevertheless, we have removed the term “valuable” from the conclusion, as suggested.

**Line 659**: “The findings of the present study provide valuable insights into the complex interplay …” **was changed** **to** “The findings of the present study provide insights into the complex interplay...”

**RESPONSES TO THE REVIEWER #2**

**General comments:** “I found this manuscript interesting, well-written and with a huge amount of data correctly analyzed. As reported by the authors, it represents the first attempt to connect trait diversity to fish landings over time through a comprehensive analysis using null models, a nice approach which led to interesting results, even if limited in some cases by the strict relation among fishing activities and ecosystems dynamics, which, as the authors know, match with multiple aspects of the anthropogenic pressures, such as climate changes, pollution, habitat removal for coastal areas, etc. However, considering the novelty of the study for the area, and the good results obtained, even this strictly focused view could result important for future studies in this field, also to deepen other aspects starting from the data here reported by Eudriano F.S. Costa and colleagues.”

Response:

Thank you for your positive feedback on our manuscript. We are pleased that you found the study interesting, well-written, and our data analysis robust. As you noted, our work represents a pioneering effort to link trait diversity to fish landings over time using null models, and we appreciate your acknowledgment of this novel approach and its intriguing results. We are aware of the complex interactions between fishing activities and ecosystem dynamics, which include various anthropogenic pressures such as climate change, pollution, and habitat removal in coastal areas. Despite these complexities, we believe that our focused approach provides valuable insights and a foundation for future research in this area. We hope that our study will encourage further investigations into these multifaceted interactions, building upon the data and findings we have presented.

**Specific comments**

**Comment 1:** “Introduction section missed an important aspect of fisheries research, linked to the use of different fishing methods and their relapses in marine environment. Indeed, by-catch represents an important source of bias in scientific data collection, because often discarded after capture without landing. At the same time, essential species for marine ecosystems are often collected within this undetected resource, leading to important consequences in habitats equilibria and their protection's management. Please add a period about this topic in this section, to contextualize better the treated topic also considering the important limitations which still today affect the landed's databases from a biological point of view.”

Response:

We agree that the issue of by-catch, which often goes unreported and discarded without landing, introduces a source of bias in this type of study. This can have critical consequences for diversity metric estimation and understanding. We recognized that this important aspect was not included in our study due to a lack of available information. To address this, we have included a discussion subhead (4.4 Study limitations and future directions) on this topic to highlight the limitations of our study and importance of considering unreported catches in future research.

**Lines 631-655**: “It is important to highlight the limitations of the present study and identify key aspects for future research. The current study lacked information on unreported catches, which means the potential effects of fishing pressure on unreported species were not investigated. Future studies should incorporate data on unreported catches to provide a more comprehensive understanding of fishing impacts on ecological roles played by all species caught. Although the Azorean fleets primarily use selective fishing gear such as hooks and lines, it is essential to consider the effects of fishing pressure on fish assemblages in a multi-gear context. Different gear types can significantly affect trait diversity [15, 81]. Additionally, the study did not evaluate the predatory approaches of the species, including their vertical migrations during feeding activities. These behaviors can significantly impact ecosystem dynamics and species interactions, so future research should examine these predatory behaviors to understand their impacts on ecosystem functioning. Furthermore, it is crucial to assess the effects of environmental factors and climate change on the traits of reported and unreported species [82]. Such changes have been reported to significantly alter essential traits, such as size and age at first maturity and fecundity over time [83]. Finally, considering life-history traits as numeric rather than categorical− for instance, size and age at first maturity, asymptotic length, growth rate, longevity, fecundity, mortality rate, larval pelagic duration, and spawning season length− would allow for the use of a wide range of statistical methods, providing deeper insights into trait diversity, ecological roles and ecosystem functioning. This approach would also offer a better understanding of the evolution of species populations under fishing and climate-induced pressures.” **was inserted**.

**Comment 2:** “Try to improve the quality of all the Figures, especially the ones that reports written informations, such as 2 and 5, which resulted very hard-to-read in the present form.”

Response:

While we appreciate the feedback on the figures, we believe that the quality of Figures 2 and 5 is adequate. The images were created using high-resolution software (R software), ensuring clarity and readability. These figures are highly informative, and presenting a large amount of essential results in a single figure in an understandable manner. Therefore, we believe that the current design effectively conveys all necessary analyses and information. We understand the importance of accessibility and will consider minor adjustments to enhance their readability further. If there are specific areas of concern within these figures, please provide detailed feedback so we can address them appropriately.

**Comment 3:** “Table 1: please add more details in the caption about the trophic position and growth coefficient values.”

Response:

**Line 185**: “^†^ It represents the position that fish occupy within their respective food webs [37].” **was inserted**.

**Lines 186-189**: “^‡^ This is a parameter of the von Bertalanffy growth function (also known as the growth coefficient), expressing the rate (1/year) at which the asymptotic length is approached. It quantifies how quickly an organism grows towards its maximum possible size over time [31].” **was inserted**.

**Comment 4:** “The period among lines 206 and 217 could results more appropriate in introduction section, due its more general view compared to the subsequent part of the paragraph which on the contrary resulted focused on the methodology of present study.”

Response:

We appreciate the reviewer's suggestion regarding the placement of the content between lines 206 and 217. However, we respectfully disagree with this recommendation. The period in question is integral to the methodological context of our study, providing specific information necessary for understanding the functional diversity metrics and their changes in response to potential fishery impacts shown in Fig. 1. Moving this content to the Introduction section, which aims to provide a broad overview of the study, might dilute the focus on our methodological framework and reduce clarity. We believe its current placement ensures a seamless transition from the general context to the specific methods employed, thus maintaining the logical flow of our manuscript.

**Comment 5:** “Line: 319: Are the authors referring as "types" to local and coastal fishing vessels reported in lines 157-158? Please clarify and better argue this first essential sentence of the results section. Considering the amount of parameters and variables (correctly) analyzed in your study, being more clear as possible in focal points could results essential for the readers.”

Response:

We appreciate the reviewer's attention to clarity and the need for precise language. Yes, the term "types" in line 319 refers to the local and coastal fishing fleets mentioned in lines 157-158. We have revised the use of fishing type:

**Line 308**: “…between the two fishing types (S1 Table)” **was changed** to “…between the two fishing fleets (**S2 Table**).”.

**Line 314**: “…concerning the fishing type” **was changed** to “…concerning the fishing fleet…”.

**Comment 6:** “Within the results section, try to reduce comments more similar to discussion than results, such as lines 354, 366 (..indicating..), 382 (..suggest..). I understand is not simple to totally separate the concepts of results and discussion in this kind of manuscript (maybe more adapt to a style as "results & discussion" section), but try your best for manuscript fluency, if possible.”

Response:

We acknowledge the challenge of completely separating results from discussion in this type of manuscript. However, we have revised the results section to minimize commentary that leans more towards discussion. Specifically, we rewritten the lines, 354, 366, and 382 to focus strictly on the presentation of data and findings. This effort should enhance manuscript’s fluency and maintain a clearer distinction between results and discussion.

**Lines 343-344**: “…functional space, indicating variations in their habitat utilization (Fig 5B).” **was changed** to “…functional space, with variations in their habitat utilization (Fig. 5B).”.

**Lines 355**: “…biplot, indicating an unclear association between...” **was changed** to “…biplot, with an unclear association between...”

**Lines 373-376**: “The scattered arrangement of the most landed species in the functional space suggest diverse functional traits related to feeding function, including variations in maximum size, trophic position, food consumption and diets (**Fig 5F**).” **was changed** to “The scattered arrangement of the most landed species in the functional space reveals a variety of functional traits associated with feeding function, encompassing variations in maximum size, trophic position, food consumption and diets (**Fig 4F**).”

**Comment 7:** “I understand the focus of this study, and agree with, but the main limitation of this document is represented by the climate changes effects occurred during the studied period all over the world, comprises the treated area, which were not mentioned at all. Indeed, even if fishing activities represents a strong source of alterations for marine organisms, a wider approach should be adopted when evaluating ecosystem equilibria, as in this case. At least mentioning as limitation of the study would be correct, highlighting the importance to cross multiple approaches to this problem. Indeed, as stated by the authors about the decline in ecosystem functions, and hypothesized from literature for the Azores (reported in line 424) "..particularly due to fishing activities", the reader is induced in thinking also to other untreated aspects, over fishing activities. Also, the period about life traits and reproductive cycle aspects (lines 593-619) couldn't ignore these additional aspects involved in environmental changes, which are reported to strongly modified essential functions, as for example: spawning, hatching and survival of teleosts.”

Response:

Indeed, climate change effects can unfold over decades, and long-term data collection would offer valuable insights into temporal trends. However, such comprehensive data gathering is often resource-intensive and requires substantial logistical support, including consistent funding and access to diverse marine habitats. To assess the effects of climate change on species traits at an intraspecific level, numerical traits sensitive to environmental shifts over decades relative to fisheries—such as length at first maturity, fecundity, growth rate, maximum size, and other life history characteristics—are essential. Ideally, these variables should have been measured annually over the past 40 years to capture any changes in these traits. Our study focuses on investigating changes in species richness and their trait combinations within the landed species in the Azores, rather than tracking intraspecific variations over time. Therefore, we acknowledge the limitations of our approach, which works within practical constraints to provide insights based on available data and methodologies. Climate change was not explicitly discussed in our study because distinguishing its effects from those of fishing pressure on life-history traits in fish populations is challenging. Future research efforts should aim to integrate multiple approaches to better understand and disentangle these complex interactions affecting marine ecosystems. Nevertheless, a paragraph highlighting the limitations of our study, as well as suggestions for future studies, has been inserted in the Discussion section.

**Lines 631-655**: “It is important to highlight the limitations of the present study and identify key aspects for future research. The current study lacked information on unreported catches, which means the potential effects of fishing pressure on unreported species were not investigated. Future studies should incorporate data on unreported catches to provide a more comprehensive understanding of fishing impacts on ecological roles played by all species caught. Although the Azorean fleets primarily use selective fishing gear such as hooks and lines, it is essential to consider the effects of fishing pressure on fish assemblages in a multi-gear context. Different gear types can significantly affect trait diversity [15, 81]. Additionally, the study did not evaluate the predatory approaches of the species, including their vertical migrations during feeding activities. These behaviors can significantly impact ecosystem dynamics and species interactions, so future research should examine these predatory behaviors to understand their impacts on ecosystem functioning. Furthermore, it is crucial to assess the effects of environmental factors and climate change on the traits of reported and unreported species [82]. Such changes have been reported to significantly alter essential traits, such as size and age at first maturity and fecundity over time [83]. Finally, considering life-history traits as numeric rather than categorical− for instance, size and age at first maturity, asymptotic length, growth rate, longevity, fecundity, mortality rate, larval pelagic duration, and spawning season length− would allow for the use of a wide range of statistical methods, providing deeper insights into trait diversity, ecological roles and ecosystem functioning. This approach would also offer a better understanding of the evolution of species populations under fishing and climate-induced pressures.” **was inserted**.

**Comment 8:** “Lines 555-576: in this context, it would be also important to evaluate the different predatory approaches of the studied species, which sometimes use also vertical migrations during their feeding activity, sometimes not. Moreover, also different fishing gears, baited or not, sometimes drives the species in moving during their feeding activity. I understand that these are not simple aspects to evaluate without specific approaches, but, at the same time, should be mentioned among the possible sources of biases.”

Response:

**Lines 631-655**: “It is important to highlight the limitations of the present study and identify key aspects for future research. The current study lacked information on unreported catches, which means the potential effects of fishing pressure on unreported species were not investigated. Future studies should incorporate data on unreported catches to provide a more comprehensive understanding of fishing impacts on ecological roles played by all species caught. Although the Azorean fleets primarily use selective fishing gear such as hooks and lines, it is essential to consider the effects of fishing pressure on fish assemblages in a multi-gear context. Different gear types can significantly affect trait diversity [15, 81]. Additionally, the study did not evaluate the predatory approaches of the species, including their vertical migrations during feeding activities. These behaviors can significantly impact ecosystem dynamics and species interactions, so future research should examine these predatory behaviors to understand their impacts on ecosystem functioning. Furthermore, it is crucial to assess the effects of environmental factors and climate change on the traits of reported and unreported species [82]. Such changes have been reported to significantly alter essential traits, such as size and age at first maturity and fecundity over time [83]. Finally, considering life-history traits as numeric rather than categorical− for instance, size and age at first maturity, asymptotic length, growth rate, longevity, fecundity, mortality rate, larval pelagic duration, and spawning season length− would allow for the use of a wide range of statistical methods, providing deeper insights into trait diversity, ecological roles and ecosystem functioning. This approach would also offer a better understanding of the evolution of species populations under fishing and climate-induced pressures.” **was inserted**.

**Comment 10:** “Add the potential limitations of this study to the conclusion section.”

Response:

The limitations of the present study and aspects to be considered in future studies have been included at the end of the Discussion. All the authors agreed that this paragraph fits better in the end of the Discussion than Conclusion.

**Lines 631-655**: “It is important to highlight the limitations of the present study and identify key aspects for future research. The current study lacked information on unreported catches, which means the potential effects of fishing pressure on unreported species were not investigated. Future studies should incorporate data on unreported catches to provide a more comprehensive understanding of fishing impacts on ecological roles played by all species caught. Although the Azorean fleets primarily use selective fishing gear such as hooks and lines, it is essential to consider the effects of fishing pressure on fish assemblages in a multi-gear context. Different gear types can significantly affect trait diversity [15, 81]. Additionally, the study did not evaluate the predatory approaches of the species, including their vertical migrations during feeding activities. These behaviors can significantly impact ecosystem dynamics and species interactions, so future research should examine these predatory behaviors to understand their impacts on ecosystem functioning. Furthermore, it is crucial to assess the effects of environmental factors and climate change on the traits of reported and unreported species [82]. Such changes have been reported to significantly alter essential traits, such as size and age at first maturity and fecundity over time [83]. Finally, considering life-history traits as numeric rather than categorical− for instance, size and age at first maturity, asymptotic length, growth rate, longevity, fecundity, mortality rate, larval pelagic duration, and spawning season length− would allow for the use of a wide range of statistical methods, providing deeper insights into trait diversity, ecological roles and ecosystem functioning. This approach would also offer a better understanding of the evolution of species populations under fishing and climate-induced pressures.” **was inserted**.

**Lines 924-927**: “81. Koutsidi M, Tzanatos E, Machias A, Vassilopoulou V. Fishing for function: the use of biological traits to evaluate the effects of multispecies fisheries on the functioning of fisheries assemblages. ICES J Mar Sci. 2016;73: 1091–1103. doi:10.1093/icesjms/fsw006” **was inserted**.

**Lines 928-930**: “82. Nagelkerken I, Allan BJM, Booth DJ, Donelson JM, Edgar GJ, Ravasi T, et al. The effects of climate change on the ecology of fishes. Yu W, editor. PLOS Clim. 2023;2: e0000258. doi:10.1371/journal.pclm.0000258” **was inserted**.

**Lines 931-934**: “83. Barausse A, Michieli A, Riginella E, Palmeri L, Mazzoldi C. Long‐term changes in community composition and life‐history traits in a highly exploited basin (northern Adriatic Sea): the role of environment and anthropogenic pressures. J Fish Biol. 2011;79: 1453–1486. doi:10.1111/j.1095-8649.2011.03139.x” **was inserted**.

**Comment 11:** “Double-check references list for scientific name and style.”

Response:

All references have been checked and adjusted to conform to the PLOS ONE style.

**Lines 790-792**: “…Quantifying Patterns in Fish Assemblages and Habitat Use Along a Deep Submarine Canyon-Valley Feature Using a Remotely Operated Vehicle. Front Mar Sci. 2021;8: 608665…” **was corrected to** “Quantifying patterns in fish assemblages and habitat use along a deep submarine canyon-valley feature using a remotely operated vehicle”.

**Line 817**: “…fishery management. Fisheries Manag Eco…” **was corrected to** “fishery management. Fisheries Manag Ecol.”.

**Authors’ corrections**

**Line 211**: “…the landed assemblage over a specific period (Villéger et al., 2008).” **was changed to** “…the landed assemblage over a specific period [51].”

**Line 344**: “.*Sparissoma cretense..*.” **was corrected** to “.*Sparisoma cretense..*.”

**Line 396**: “…significance between local and costal pair…” **was changed to** “…significance between local and coastal pair…”

**Lines 935-995**: Captions for your Supporting Information files were included.

**Table 1**: “102” **was corrected to** “103”

**Figure**: “Fig 1” **was removed** due to copyright requirements.

**Line 127**: “…ecosystem along the Mid-Atlantic Ridge (**Fig 1**).” **was changed to** “…ecosystem along the Mid-Atlantic Ridge.”

**Lines 133-134**: “**Fig 1**. Map of the Azores archipelago displaying its islands and Economic Exclusive Zone (dotted lines). Map was created using QGIS (version 3.21.2- Tisler)” **was removed**.

**Line 203**: “…trait values (**Fig 2**) [50].” **was changed to** “…trait values (**Fig 1**) [50].”

**Line 213**: “… (**Fig 2A**).” **was changed to** “… (**Fig 1A**).”

**Line 215**: “… (**Fig 2B**).” **was changed to** “… (**Fig 1B**).”

**Line 219**: “… (**Fig 2B**).” **was changed to** “… (**Fig 1C**).”

**Line 224**: “… (**Fig 2D**).” **was changed to** “… (**Fig 1D**).”

**Line 227**: “…**Fig 2**. Conceptual diagrams illustrating the functional diversity metrics.” **was changed to** “…**Fig 1**. Conceptual diagrams illustrating the functional diversity metrics…”.

**Lines 311-312**: “…for both fishing fleets (**Figs 3A and 4A**).” **was changed to** “…for both fishing fleets (**Figs 2A and 3A**).

**Line 318**: “…between 2000s and 2010s (**Fig 3B**)....” **was changed to** “…for between 2000s and 2010s (**Fig 2B**)…”.

**Line 320**: “…respectively (**Fig 3C**).” **was changed to** “…respectively (**Fig 2C**)...”

**Line 321**: “…different decades pairs (**Fig 3D**).” **was changed to** “…different decades pairs (**Fig 2D**).”

**Line 323**: “…and 00s-10s (**Fig 4B**).” **was changed to** “…and 00s-10s (**Fig 3B**).”

**Line 324**: “…across functions and decades (**Figs 4C-4D**).” **was changed to** “…across functions and decades (**Figs 3C-3D**).”

**Line 326**: “…(**Fig 4C**).” **was changed to** “…(**Fig 3C**).”

**Line 342**: “…bathydemersal species (**Fig 5A**).” **was changed to** “…bathydemersal species (**Fig 4A**).”

**Line 344**: “…(**Fig 5B**).” **was changed to** “…(**Fig 4B**).”

**Line 346**: “…other highly landed species (**Fig 5B**).” **was changed to** “…other highly landed species (**Fig 4B**).”

**Line 354**: “…axis 1 (**Fig 5C**).” **was changed to** “…axis 1 (**Fig 4C**).”

**Line 357**: “…*Lepidopus caudatus* (**Fig 5D**).” **was changed to** “…*Lepidopus caudatus* (**Fig 4D**).”

**Line 359**: “…vertex species (**Fig 5D**).” **was changed to** “…vertex species (**Fig 4D**).”

**Line 370**: “…with the FCA axis 2 (*r^2^* > 0.55) (**Fig 5E**).” **was changed to** “…with the FCA axis 2 (*r^2^* > 0.55) (**Fig 4E**).”

**Line 370**: “…food consumption and diets (**Fig 5F**).” **was changed to** “…food consumption and diets (**Fig 4F**).”

**Line 375**: “…traits (**Fig 5F**).” **was changed to** “…traits (**Fig 4F**).”

**Line 386**: “…less than 0.54/ year (**Fig 5G**).” **was changed to** “…less than 0.54/ year (**Fig 4G**).”

**Line 388**: “…of the functional space (**Fig 5H**).” **was changed to** “…of the functional space (**Fig 4H**).”

**Line 388**: “…most landed species (**Fig 5H**).” **was changed to** “…most landed species (**Fig 4H**).”

**Line 399**: “**Fig 3.** Functional diversity metrics…” **was changed to** “**Fig 2.** Functional diversity metrics…”

**Line 409**: “**Fig 4.** The y-axis p-values indicate…” **was changed to** “**Fig 3.** The y-axis p-values indicate…”

**Line 418**: “**Fig 5.** Exploring the relationships between…” **was changed to** “**Fig 4.** Exploring the relationships between…”

**Supplementary information.** “Supplementary Table 6” **was corrected to** “S7 Table”

**Supplementary information.** “Supplementary Table 8” **was corrected to** “S9 Table”

**Supplementary information - S2 Figure.** “…in blue can be seen in **Supplementary Table 8**.” **was corrected to** “…in blue can be seen in **S9 Table**.”
